# Supplementary material for: The consumption of alcohol by adolescent schoolchildren: Differences in the triadic relationship pattern between rural and urban environments
Source: PLoS One. 2020 Nov 2;15(11):e0241135. doi: 10.1371/journal.pone.0241135 (PMC7605695; doi:10.1371/journal.pone.0241135)
Supplement: Attachment — Submitted filename: Response to Reviewers.pdf [file pone.0241135.s004.pdf]

Reviewer 1

Reviewer #1: Recommendation: Minor Revisions

Thank you for the opportunity to review for PLoS One. Thank you to the authors for their hard work and interesting study. Overall, there might be a relevant and important paper here, however some sections are unclear and need refining if they are to sell the novelty of the paper fully. I will note that I am not an expert on social network analysis (I only have a basic quantitative understanding of the specific methodologies involved) and the paper should be considered by a relevant statistician before a decision is made.

Strengths

- There are few empirical social network analyses out there due to the difficulty in obtaining data suggesting a potential novel study design

- Potentially interesting analyses on the network structure and how varies by some basic sociodemographic issues

- Well written discussion

We thank reviewer one for his appreciation of the research. We are very happy to know that you find our research interesting. We hope to be able to satisfy all the proposals and comments you indicate in this review.

Areas for Improvement

- Line 39: 'In this sense, different research' doesn't quite work with the rest of the statement, suggest revise

We have changed "different research" to "some studies".

- Line 44: Drop use of 'extremely' as no need to overplay the importance of the issue

It has been removed.

- Lines 94-94: Repeated use of (i) for different points is confusing to the reader and suggests overlap when there is not. Please revise.

It has been changed to (1) and (2) in the second phrase. We hope it's not confusing anymore.

- The introduction is not structured effectively and jumps about a lot. It would benefit from thinking about the flow of ideas and the content being discussed. It also doesn't feel like there is much novelty here. I am sure there is, but it is not explained clearly in particular that the premise of the study appears to suggest that we already know a lot of this.

We have modified the introduction. We hope that after the proposed changes that have been made in this section, we will have fulfilled this requirement as well.

- Line 102: What is 'DE', please define in first use

Sorry, DE is the English translation of SD (Standard Deviation). It has been changed.

- Line 149: ‘We analysed the la existence’ needs correcting

It has been corrected.

- Line 153: Unsure what is the point of the statistical test here as the numbers appear to be comparing whether there is a difference between risk and no risk in women. Although a ‘statistically insignificant’ effect is reported, there is a <1% difference here which suggests in reality it isn’t important. I would have expected a comparison of the metric between women and men here instead (i.e. were women more or less at risk compared to men) - this might be what you did but it isn’t clear that it is.

We have modified the manuscript with the following sentence:

“The risk consumption of alcohol is significantly seen to be associated with the female gender, in which we observed that 50.48% of women has a risk alcohol consumption **compared to 49.52% of men who were at risk of alcohol consumption** (50.48 Vs 49.52%; X^2=4.40; p=0.036).”

- There are many results to report meaning the section is rather brief. I think this is more an issue in how they are discussed and some of their novelties are not teased out. It might need greater clarity especially to help guide a lay reader which will be important to increase the awareness and reach of your paper.

We have modified the discussion. We hope that after the proposed changes that have been made in this section, we will have fulfilled this requirement as well.

- No discussion of limitations of research in the discussion. What were the major weaknesses or opportunities for future research? How representative were your data? How might you improve on your study? What data could you have collected to make it stronger? We would normally expect some reflection over the strengths and weaknesses of a paper.

We have modified the discussion. We hope that after the proposed changes that have been made in this section, we will have fulfilled this requirement as well.

- Data and analytical code are not openly available. If possible (more for the former than the latter which should be fine), this should be made available to adhere to PLoS One guidance. This will also improve the impact of your paper.

Attending to this suggestion, we have uploaded the questionnaires used at zenodo https://doi.org/10.5281/zenodo.3965836 and all database is available at zenodo https://doi.org/10.5281/zenodo.3968264 .

We hope that after all the proposed changes that have been made, we will have fulfilled this requirement as well.

Reviewer 2

Reviewer #2: Thank you for the opportunity to the manuscript entitled, The consumption of alcohol by adolescent schoolchildren: Differences in the triadic relationship pattern between rural and urban environments submitted to PLOS ONE (PONE-D-20-13204). The study examines alcohol use among a sample of adolescents to better understand the geographic contribution to alcohol consumption. The study also examines the influence of peer groups by assessing triadic relationships. The manuscript has significant grammatical errors and needs copyediting. The introduction is brief and needs additional discussion on the contribution of geographic area on alcohol availability and alcohol consumption. The methods section is most problematic; there are essential details that are missing that limit interpretation of the results. In its current form, the manuscript is not ready for publication. Specific comments are listed below.

We thank reviewer one for his appreciation of the research. We are very happy to know that you find our research interesting. We hope to be able to satisfy all the proposals and comments you indicate in this review.

Line 37, define risk limits.

It has been defined in the manuscript:

"alcohol to risk limits *(a value established by the WHO in which alcohol is defined as having a very harmful effect on health)* [2]."

Line 38 should be supported by additional citations. Similarly, line 47 should be supported by additional citations. The authors should review the literature on neighborhood environment (and rural/urban) and alcohol availability and consumption.

We have added more references to these paragraphs.

The language is too causal in some areas "it is important that nowadays"

We have hired a native English translator to review the entire manuscript. We hope this version will be more readable.

Line 57 should be rewritten

Line 89 should be rewritten

The last paragraph is vague. The authors should be specific when discussing the aims (e.g. including the outcome measure).

We have rewritten lines 57, 89 and the last paragraph of the introduction. In addition, this section was modified following the suggestions of reviewer 1.

Are state schools similar to public schools in the US?

Yes, state schools are the same as public schools. We've changed the terminology used.

How was rural and urban defined?

We have added this text to the manuscript:

“The difference between rural and urban is defined by the proportion of the population. Those nuclei with a population of more than 50,000 are considered to be urban areas [26,27]”.

Differences in alcohol availability?

We have added this text to the manuscript:

“It should be noted that the rural areas being more isolated had less access to various drugs [28,29].”

Were adolescents under 18 able to consent? Was there parental consent?

We have added this text to the manuscript:

“The initial population was 270 adolescents, 195 of whom signed the informed consent form or their parents in the case of adolescents under 18.”

What does “The recruitment was incidental” mean?

We have added this text to the manuscript:

“that is, incorporation into the study was voluntary, without bias of any kind.”

Response rate? Compensation for completing the assessment?

We have added this text to the manuscript:

“The initial population was 270 adolescents, 195 of whom signed the informed consent form or their parents in the case of adolescents under 18.”

“ad-hoc” ones is too casual. This sentence needs a citation. Which validated instruments were used?

We have changed the word “ones” and we have added two references and we have changed the text to:

“, by means of validated instruments (AUDIT test questionnaire) and other “ad-hoc” ones to cover the interest of the research [27,28]”

How many questions were included? How was the outcome measure defined?

In relation to the first question, we have added this text to the manuscript:

“The complete questionnaire used is available at : https://doi.org/10.5281/zenodo.3965836”

With regard to the “outcome measure”, the Alcohol Use Disorder Identification Test (AUDIT) is composed of ten questions that explore three domains: risk consumption, symptoms of dependence, and harmful consumption. Each question is scored from 0 to 4. Cherpitel [https://doi.org/10.1016/0376-8716(95)01199-4] proposes cut-off points to identify risk consumption, harmful consumption and dependence. Using the cut-off point at 8 for men and 6 for women, AUDIT is a sensitive test (51-97%) for detecting harmful alcohol use, abuse or dependence. It is currently the most recommended risk screening technique, based on its simplicity of application and its focus in the recent past (last year).

How were schools and areas selected? The year(s) of data collection should be included.

We have added this text the manuscript:

“The selection was carried out taking into account the receptivity to the project by the centres, the relations of the research team with the directors and teachers, and the search for educational centres with which to obtain a sample of similar size, which would allow us to compare the rural and urban areas.”

And we have added this text including the year:

"… attending rural and urban state public schools in the area of El Bierzo (León, Spain), *during the 2015-2016 academic year.”*

Inclusion and exclusion criteria?

We have added this text to the manuscript:

Since not having the informed consent signed by the parents was considered a criterion for exclusion.

The data analysis section should be rewritten. Alpha should be less than 0.05, not less than or equal to.

This was a transcription error, the criterion of $p<0.05$ was used. Therefore, the analysis performed was correct. We have changed it in the manuscript.

Any control variables?

We have added this text to the manuscript:

Age was used as a control variable, being between 16 and 19 years old.

What statistical test was used?

We have added this text to the manuscript:

“To determine correlations, we used Pearson’s correlation coefficient.”

Any missing data?

No data were lost because all participants filled in all the questions.

Power analysis?

The power analysis has been carried out with the program G*Power 3.1.9.6 and the values obtained in table 4 have been added.

There are areas in the results that are not English “la existence” and “consumption de alcohol.”

We have corrected these mistakes.

Line 160 belongs in the methods

We have moved this part sentence to methods.

Table 4 does not stand alone and is difficult to interpret.

We have modified the names of the "classrooms" that were in Spanish. We hope that now the table is more understandable.

The results cannot be fully evaluated without the missing information in the methods section.

We hope that after all the proposed changes that have been made, we will have fulfilled this requirement as well.
