# Supplementary material for: The consumption of alcohol by adolescent schoolchildren: Differences in the triadic relationship pattern between rural and urban environments
Source: PLoS One. 2020 Nov 2;15(11):e0241135. doi: 10.1371/journal.pone.0241135 (PMC7605695; doi:10.1371/journal.pone.0241135)
Supplement: Attachment — Submitted filename: PONE-D-20-13204.docx [file pone.0241135.s003.docx]

Thank you for the opportunity to the manuscript entitled, *The consumption of alcohol by adolescent schoolchildren: Differences in the triadic relationship pattern between rural and urban environments* submitted to PLOS ONE (PONE-D-20-13204). The study examines alcohol use among a sample of adolescents to better understand the geographic contribution to alcohol consumption. The study also examines the influence of peer groups by assessing triadic relationships. The manuscript has significant grammatical errors and needs copyediting. The introduction is brief and needs additional discussion on the contribution of geographic area on alcohol availability and alcohol consumption. The methods section is most problematic; there are essential details that are missing that limit interpretation of the results. In its current form, the manuscript is not ready for publication. Specific comments are listed below.

Line 37, define risk limits.

Line 38 should be supported by additional citations. Similarly, line 47 should be supported by additional citations. The authors should review the literature on neighborhood environment (and rural/urban) and alcohol availability and consumption.

The language is too causal in some areas “it is important that nowadays”

Line 57 should be rewritten

Line 89 should be rewritten

The last paragraph is vague. The authors should be specific when discussing the aims (e.g. including the outcome measure).

Are state schools similar to public schools in the US? How was rural and urban defined? Differences in alcohol availability?

Were adolescents under 18 able to consent? Was there parental consent?

What does “The recruitment was incidental” mean?

Response rate? Compensation for completing the assessment?

“ad-hoc” ones is too casual. This sentence needs a citation. Which validated instruments were used?

How many questions were included? How was the outcome measure defined?

How were schools and areas selected? The year(s) of data collection should be included.

Inclusion and exclusion criteria?

The data analysis section should be rewritten. Alpha should be less than 0.05, not less than or equal to. Any control variables? What statistical test was used? Any missing data? Power analysis?

There are areas in the results that are not English “la existence” and “consumption de alcohol.”

Line 160 belongs in the methods

Table 4 does not stand alone and is difficult to interpret.

The results cannot be fully evaluated without the missing information in the methods section.
