# Supplementary material for: The consumption of alcohol by adolescent schoolchildren: Differences in the triadic relationship pattern between rural and urban environments
Source: PLoS One. 2020 Nov 2;15(11):e0241135. doi: 10.1371/journal.pone.0241135 (PMC7605695; doi:10.1371/journal.pone.0241135)
Supplement: Attachment — Submitted filename: Response to Reviewers.docx [file pone.0241135.s005.docx]

**Reviewer 1**

Reviewer #1: All changes made and the paper is stronger for it. Thank you for your hard work, and well done. Accept.

We appreciate the review and are pleased to know that all suggestions have been properly addressed.

**Reviewer 2**

Reviewer #2: Reviewer #2: Thank you for the opportunity to review the revised manuscript entitled The consumption of alcohol by adolescent schoolchildren: Differences in the triadic relationship pattern between rural and urban environments submitted to PLOS One (PONE-D-20-13204R1). The manuscript has improved but there remains significant problems, mainly related to the language and grammar. Specific comments are below.

We appreciate your review and are pleased to learn that the manuscript has improved following the changes made. We hope to be able to satisfy the new comments indicated in this revision.

The entire abstract needs copyediting. Adolescence is confused with adolescents. Analyse should be analyze. The methodology section should be expanded. Results from the regression models should be added to the abstract.

Taking into account your suggestions, we have hired a translator who has been responsible for the copyediting of the entire manuscript.

In the manuscript "with tracked changes" it is possible to see the modifications made by the translator.

In addition, we have modified the abstract by adding more information, as suggested.

Similarly, the introduction needs significant copyediting.

The translator performed this task. We hope that the section will be better written.

The sample population and recruitment should start the methods section. Was assent obtained for minors?

Following this suggestion, we have added this information to the manuscript in the first paragraph of section 2.

What does the initial population refer to (students that expressed interest)? What geographic unit/size for classifying urban/rural, mentioning 50,000 population alone is insufficient. Nuclei? A better description of access between rural and urban areas would be appreciated.

In response to this suggestion, we have added more justified information with scientific references.

“The total population of the different institutes was 270 adolescents. Of these, 195 adolescents or their parents in the case of adolescents under 18 years of age signed the informed consent.

In this way, the sample was made up of 195 adolescents aged between 16 and 19, attending rural and urban public schools in the area of El Bierzo (León, Spain), during the 2015-2016 academic year. The selection was with the consent of the project from the centers. We also took into account the relationships of the research team with the directors and professors, which facilitated the launch of the research. A search for educational centers in rural and urban settings was carried out in order to obtain a sample of similar size, which would allow us to compare both rural and urban areas.

There are various definitions for characterising rural settings. The Ministry of the Environment defines the proportion and density of the population as the most used criterion. In Spain, rural areas are defined as "A geographic space comprising the aggregation of municipalities or smaller local entities with a population <30,000 inhabitants and a density <100 inhabitants per Km ^2^ [26].”

The target population described at the end of the first paragraph on page 6 should be moved to the beginning of the methods section and this is unclear.

In line with this suggestion, the text has been amended and moved to the position indicated.

The results of the power analysis should be mentioned in the text. The analyses should be reviewed by a statistician. The entire methods section needs copyediting.

We have included a comment about the power analysis in the text. The results of the statistical analysis have been revised and reported in the text as indicated by the statistician.

The study overall is not as strong as it could be. The sample size is relatively small and the analyses are not well described.

We hope that after all the proposed changes that have been made, we will have fulfilled this requirement as well.
