# Supplementary material for: The consumption of alcohol by adolescent schoolchildren: Differences in the triadic relationship pattern between rural and urban environments
Source: PLoS One. 2020 Nov 2;15(11):e0241135. doi: 10.1371/journal.pone.0241135 (PMC7605695; doi:10.1371/journal.pone.0241135)
Supplement: Attachment — Submitted filename: Response to Reviewers.docx [file pone.0241135.s006.docx]

**Additional Editor Comments (if provided):**

The manuscript will benefit from copy editing. It would be helpful to seek help from the native English speaker.

First of all, I would like to thank the reviewers for their work. We are pleased to have solved all the suggestions indicated in the two reviews by the two reviewers.

Following the suggestions of the reviewers and the editor, we have sent our article to the company American Journal Experts (AJE) for copyediting. I have attached the certificate of review with the code TL4M4QZ6 in the inventory of files of the journal.

**Reviewer 1**

Reviewer #1: I had not requested any further comments so all good on my side and wishing you the best of luck with your paper.

We are glad to know that we have taken care of all your suggestions correctly. Thank you very much for your message.

**Reviewer 2**

Reviewer #2: The authors have addressed the comments. Grammar and readability continues to be a problem. Methodology/analyses appropriate.

We are glad to know that we have taken care of all your suggestions correctly. Thank you very much for your message.

Following your suggestions, we have sent our manuscript to the company American Journal Experts (AJE) for copyediting.
